# Antidiarrhoeal, antisecretory and antispasmodic activities of *Matricaria chamomilla* are mediated predominantly through K^+^-channels activation

**DOI:** 10.1186/s12906-015-0595-6

**Published:** 2015-03-24

**Authors:** Malik Hassan Mehmood, Siraj Munir, Uzair Ali Khalid, Mudassir Asrar, Anwarul Hassan Gilani

**Affiliations:** Natural Product Research Division, Department of Biological and Biomedical Sciences, Faculty of Health Sciences, Medical College, The Aga Khan University, Stadium Road, P.O. Box 3500, Karachi, 74800 Pakistan; Department of Botany, University of Balochistan, Quetta, Pakistan

**Keywords:** Chamomile, Antidiarrhoeal, Antisecretory, Antispasmodic, K^+^ channels activation, Ca^++^ antagonist

## Abstract

**Background:**

*Matricaria chamomilla* commonly known as “Chamomile” (Asteraceae) is a popular medicinal herb widely used in indigenous system of medicine for a variety of ailments. However, there is no detailed study available showing its effectiveness in hyperactive gut disorders like, abdominal colic and diarrhoea. This study was designed to determine the pharmacological basis for the folkloric use of *Matricaria chamomilla* in diarrhoea.

**Methods:**

The crude aqueous-methanolic extract of *Matricaria chamomilla* (Mc.Cr) was studied for its protective effect in mice against castor oil-induced diarrhoea and intestinal fluid accumulation. The isolated rabbit jejunum was selected for the *in-vitro* experiments using tissue bath assembly coupled with PowerLab data acquisition system.

**Results:**

Oral administration of Mc.Cr to mice at 150 and 300 mg/kg showed marked antidiarrhoeal and antisecretory effects against castor oil-induced diarrhoea and intestinal fluid accumulation, simultaneously, similar to the effects of cromakalim and loperamide. These effects of plant extract were attenuated in animals pretreated with K^+^ channel antagonist, glibenclamide (GB) or 4-aminopyridine (4-AP). When tested in isolated rabbit jejunum, Mc.Cr caused a dose-dependent (0.3-3 mg/ml) relaxation of spontaneous and low K^+^ (25 mM)-induced contractions, while it exhibited weak inhibitory effect on high K^+^ (80 mM). The inhibitory effect of Mc.Cr on low K^+^-induced contractions was partially inhibited in the presence of GB, while completely blocked by 4-AP. Cromakalim, an ATP-sensitive K^+^ channel opener, caused complete relaxation of low K^+^-induced contractions with little effect on high K^+^. Pretreatment of tissues with GB blocked the inhibitory effects of cromakalim on low K^+^, while the presence of 4-AP did not alter the original effect. Verapamil, a Ca^++^ channel antagonist, caused complete relaxation of both low and high K^+^-induced contractions with similar potency. The inhibitory effect of verapamil was insensitive to GB or 4-AP. When assessed for Ca^++^ antagonist like activity, Mc.Cr at high concentrations caused rightward shift in the Ca^++^ concentration-response curves with suppression of the maximum response, similar to the effect of verapamil, while cromakalim did not show similar effect.

**Conclusions:**

This study indicates that *Matricaria chamomilla* possesses antidiarrhoeal, antisecretory and antispasmodic activities mediated predominantly through K^+^-channels activation along with weak Ca^++^ antagonist effect.

## Background

*Matricaria chamomilla* L. (synonym: *Matricaria recutita*) commonly known as Chamomile or German Chamomile, belongs to family Asteraceae [1]. Multiple species of Chamomile are spread over Europe, North-west Asia, North America, North Africa and the temperate regions of Asia [2,3]. In Pakistan, it is known as babuna or piunphulli and grows naturally in highlands of Balochistan such as, Hanna valley, Maslakh range, Muslimbagh, Kalat, Nushki, Kharan, Chaman and Ziarat. This plant is widely recognized and is more popular in the western culture for its diverse medicinal uses [1,4]. Since ancient times and in accordance with the references written by Hippocrates, Galen and Asclepius, its different parts including flowers, roots and oil have been used to treat variety of ailments [5].

Chamomile as a whole plant has been used traditionally in different forms for the treatment of multiple medical complaints such as, common cold, bronchitis, gastrointestinal spasms, epilepsy, hypertension, neuralgia, toothache, dysmenorrhea, eczema, impetigo, indigestion, colic and diarrhea [1,4-9]. Its flowers are also used as carminative and antipyretic, while its oil has been used in rheumatism, flatulence and colic [10]. In addition, this plant had a very long history of its medicinal use in ancient Egypt, Greece and Rome [11]. Chamomile is also one of the effective ingredients of several traditional formulations in the Unani and Homeopathy systems of medicine [11-15].

Phytochemical studies revealed the presence of alpha-bisabolol, cis-spiroethers sesquiterpenes (anthecotulid), cadinene, farnesene, furfural, spathulenol, and proazulene (matricarin and matricin) as plant constituents [5,16]. In addition, Chamomile has also been found to possess around 8% of flavone glycosides (apigenin 7-glycoside and its 6’-acetylated derivative) and flavonols (luteolin glucosides, quercetin glycosides, and isohamnetin); 10% of mucilage polysaccharides; 0.3% of choline and 0.1% of coumarins (umbelliferone and its methyl ether, herniarin). The presence of tannin in chamomile has also been detected less than 1% [17-19].

Pharmacological investigations showed that *Matricaria chamomilla* possesses antiinflammatory [20], antispasmodic [21,22], antibacterial [23], digestive [24], antioxidant and antidiabetic [25,26] activities. However, there is little evidence available to its medicinal use in diarrhoea. This study has been planned to explore the scientific basis for the medicinal use of *Matricaria chamomilla* in hyperactive gut disorders, like diarrhoea and abdominal spasms using different *in vivo* and *in vitro* assays.

## Methods

### Plant material and extraction

The aerial parts of *Matricaria chamomillla* L. collected and identified by Dr. Mudassir Asrar (University of Balochistan, Quetta, Pakistan). A voucher specimen # BGUOB-187 was deposited in the herbarium of Department Botany, University of Balochistan. The dried plant material weighing 400 g was used for extraction and soaked in 70% methanol for three days, with occasional shaking. The soaked material was filtered through a muslin cloth and then through a Whatman qualitative grade 1 filter paper. This procedure was repeated three times and the combined filtrate was evaporated using rotary evaporator to get the final aqueous-methanolic extract of *Matricaria chamomillla* (Mc. Cr), yielding 15% w/w [27].

### Drugs and animals

Potassium chloride, verapamil hydrochloride, loperamide and 4-aminopyridine were purchased from Sigma Chemicals Co, St Louis, MO, USA. Cromakalim and glibenclamide were purchased from Tocris, Ellisville, MO and RBI Chemicals Co, Natick, MA, USA respectively. Castor oil was purchased from Karachi chemical industries (Pvt.) Ltd. F/25 S. I. T. E., Karachi (Pakistan). All chemicals used were of the analytical grade available and solubilized in distill water/saline except cromakalim and glibenclamide, which were dissolved in 10% DMSO to prepare stock solutions. The vehicle used for solubilization was found inert on isolated tissue preparations in control experiments. Stock solutions of all chemicals were made fresh in normal saline on the day of the experiment.

BALB/c mice (weighing 20–25 g) and locally bred rabbits (weighing 1–1.5 kg) of both genders, were housed at the Animal House of Aga Khan University under controlled environmental conditions (23–25°C). The animals were fasted for 12–16 h before the experiment, whereas they were given tap water and standard diet routinely. Experiments were performed with the rulings of the Institute of Laboratory Animal Resources, Commission on Life Sciences, National Research Council [28] and also in accordance with Institutional guidelines. The study protocol (016-ECACU-BBS-13) was approved by the Ethics Committee for Animal Care and Use (ECACU) of the Aga Khan University.

### *In-vivo* assays

#### Antidiarrhoeal activity

Mice (20–25 g, n = 78) of either gender were fasted for 12–16 h before the experiment. The animals were housed in individual cages and divided into 13 groups, each containing six animals. The first group received saline along with solubilizing vehicle (10 ml/kg) orally (p.o.), acted as negative control. The group 2 and 3 received cromakalim (10 mg/kg, p.o.) and loperamide (10 mg/kg, p.o.), respectively, serving as the positive controls. The group 4 and 5 received different doses (150 and 300 mg/kg, p.o.) of Mc. Cr. Of the remaining eight groups, the group 6, 8, 10 and 12 were pretreated with glibenclamide (GB, 3 mg/kg, i.p.) and the group 7, 9, 11 and 13 were administered 4-aminopyridine (4-AP, 5 mg/kg, i.p.) 30 min before re-administration of cromakalim, loperamide or Mc. Cr. After 1 h of respective treatments, each animal received 10 ml/kg castor oil orally through a feeding needle. After 5 h, the mouse cages were individually inspected for mean defecation per group, mean number of wet feces and mean number of dry feces. The percentage protection in each group was calculated as, (mean number of dry feces/mean defecation) × 100 [29].

### Intestinal fluid accumulation

By following previously described method with slight modification [30], 12–16 h fasted mice (weighing 20–25 g, n = 84) of either gender were housed in cages in fourteen equally divided groups (n = 6). The first two groups received saline in a solubilizing vehicle (10 ml/kg, p.o.) and acted as negative controls. The animals in group 3 and 4 were administered cromakalim (10 mg/kg) and loperamide (10 mg/kg) intraperitoneally (i.p.), respectively, using a detachable U-100 insulin syringe with a 25 G × 1″ (0.50 × 25 mm) needle, as the positive controls. The group 5 and 6 were treated with increasing doses of the plant extract (150 and 300 mg/kg, p.o.). The group 7, 9, 11 and 13 were pretreated with glibenclamide (GB, 3 mg/kg, i.p.), while the group 8, 10, 12 and 14 were pretreated 4-aminopyridine (4-AP, 5 mg/kg, i.p.) 30 min before re-administration of cromakalim, loperamide or Mc. Cr. After 1 h of the respective treatments, each animal received castor oil (10 ml/kg. p.o.), except the animals in group one. All the animals were sacrificed 30 min later by cervical dislocation and the intestine was dissected out carefully, not allowing any intestinal fluid to leak out, and weighed. The results were expressed as (*Pi/Pm*) × 1000 whereas, *Pi* is the weight of the intestine and *Pm* is the weight (in g) of the animal.

### Acute toxicity test

A total of 40 Balb/c mice (20–25 g) of either sex were equally divided into four groups. The test was performed using increasing doses of the crude extract of the *Matricaria chamomillla* (1, 3 and 5 g/kg) given orally in 10 ml/kg control vehicle to different animals serving as the test groups. Another group of mice was administered control vehicle (10 ml/kg) orally as the negative control. The animals were allowed food and water *ad libitum* and kept under regular observation for 6 h to observe their piloerection, changes in exploratory behavior and blindness, while lethality was monitored up to 24 h.

### *In-vitro* experiments

#### Antispasmodic activity

The spasmolytic activity of the test material was studied by using isolated rabbit jejunum preparations [31]. Respective segments of 2 cm in length were suspended individually in 10 ml tissue baths containing Tyrode’s solution, aerated with a mixture of 95% oxygen and 5% carbon dioxide (carbogen) and maintained at 37°C using thermo-circulator. The composition of the Tyrode’s solution in mM was: KCl 2.68, NaCl 136.9, MgCl_2_ 1.05, NaHCO_3_ 11.90, NaH_2_PO_4_ 0.42, CaCl_2_ 1.8 and glucose 5.55 (pH 7.4). Intestinal responses were recorded isotonically using Bioscience Transducers coupled with PowerLab data acquisition system (ADInstruments, Sydney, Australia). Each tissue was allowed to equilibrate for at least 30 min before the addition of any drug and then stabilized with a sub-maximal concentration of acetylcholine (ACh, 0.3 μM) and the bath fluid was subsequently replaced with normal Tyrode’s solution before starting the experiment.

The myorelaxant effect of the plant extract was assessed on spontaneously contracting isolated rabbit jejunum. For elucidation of the possible mechanism of spasmolytic effect, low K^+^ (25 mM) and high K^+^ (80 mM) were used to depolarize the isolated tissues which in turn produced sustained contractions, which are considered useful for determining the different inhibitory mechanisms like, K^+^ channel activation [32] and Ca^++^channel blockade [33], respectively. The test material was then added in a cumulative fashion to obtain concentration-dependent inhibitory responses. The relaxation of isolated tissue preparations was expressed as percent of the control response mediated by added low or high K^+^-induced concentrations.

To know the nature of K^+^ channels involved in the inhibitory effect of the test material, the spasmolytic effect of the test material was studied on low K^+^-induced contractions in the absence and presence of glibenclamide (GB), an ATP-dependent K^+^-channel blocker [34] or 4-aminopyridine (4-AP), a voltage-dependent K^+^ channel blocker [35].

To confirm the presence of Ca^++^ antagonist like activity in the test material, the tissue were allowed to stabilize in normal Tyrode’s solution, which was then replaced with Ca^++^-free Tyrode’s solution containing EDTA (0.1 mM) for 30 min in order to chelate Ca^++^ from the environment and the tissues [36]. This solution was further replaced with K^+^-rich and Ca^++^-free Tyrode’s solution with following constitution (mM): KCl 50, NaCl 91.04, MgCl_2_ 1.05, NaHCO_3_ 11.90, NaH_2_PO_4_ 0.42, glucose 5.55 and EDTA 0.1. After an incubation period of 30 min in normal Tyrode’s solution, the concentration-response curves (CRCs) of Ca^++^ were constructed. When the control CRCs of Ca^++^ were found super imposable (usually after two cycles), then the tissue was pre-incubated with test substance for 60 min to confirm the possible Ca^++^ antagonist like activity [37]. In the presence of different concentrations of the test material, the CRCs of Ca^++^ were reconstructed. Non-parallel rightward shift with suppression of the maximum response of Ca^++^ in the presence of test substance indicates Ca^++^ antagonist-like antispasmodic activity.

#### Statistical analysis

All the data expressed are mean ± standard error of mean (s.e.m., n = number of experiments) and the median effective concentrations (EC_50_ values) with 95% confidence intervals (CI). The concentration-response curves were analyzed by non-linear regression (Graph-PAD program, Graph-PAD, San Diego, CA, USA). Unpaired *t-*test or One-way ANOVA followed by Dunnett’s test was used for antidiarrhoeal and intestinal fluid accumulation assays. Probability of less than 0.05 was considered significantly different.

## Results

### Effect of *Matricaria chamomillla* on castor oil-induced diarrhoea in mice

Oral administration of *Matricaria chamomillla* exhibited dose-dependent inhibitory effect against castor oil-induced diarrhoea in mice by producing 41.4 and 61.9% protection at the respective doses of 150 and 300 mg/kg. The positive controls of cromakalim and loperamide caused 65.2 and 77.2% protection, respectively, while saline treated group showed only 12.4% protection. When the effect of Mc. Cr was restudied in mice pretreated with GB or 4-AP, it was attenuated to 34.1 and 22.5% vs. 41.4% (effect of Mc. Cr without antagonist), respectively, at 150 mg/kg, and decreased to 44.9 and 29.8% vs. 61.9% at 300 mg/kg. Similarly, the effect of cromakalim was significantly reduced to 25.6% vs. 65.2% when reproduced in the presence of GB, while remained devoid of any significant change in the presence of 4-AP. Loperamide did not show sensitivity to GB or 4-AP (Table 1).

**Table 1:** **Antidiarrhoeal effect of the crude extract of** ***Matricaria chamomilla*** **(Mc.Cr) against castor oil (10 mL/kg)-induced diarrhoea in mice without and with pre-administration of 3 mg/kg glibenclamide (GB) or 5 mg/kg 4-aminopyridine (4-AP)**

**Group no.**	**Treatment**	**Dose (mg/kg)**	**Mean defecation/group**	**Mean no. of wet feces/group**	**Mean no. of dry feces/group**	**% Protection**
1	Castor oil (10 mL/kg) + Saline	10	8.92 ± 1.24	7.81 ± 2.11	1.11 ± 0.21	12.4%
2	Castor oil + Cromakalim	10	6.67 ± 0.90^**#**^	2.32 ± 0.40^**###**^	4.35 ± 1.70^**#**^	65.2%
3	Castor oil + Loperamide	10	4.70 ± 0.90^**###**^	1.07 ± 0.30^**###**^	3.63 ± 0.90^**#**^	77.2%
4	Castor oil + Mc.Cr (p.o)	150	5.47 ± 1.37**	3.20 ± 0.50**	2.27 ± 0.58*	41.4%
5	Castor oil + Mc.Cr (p.o.)	300	4.20 ± 0.29***	1.61 ± 0.92***	2.59 ± 0.33*	61.7%
**Effect of different treatments in the presence of GB or 4-AP**						
6	Castor oil + GB + Cromakalim	3 + 10	7.40 ± 0.95^$^	5.50 ± 0.70^$$$^	1.90 ± 0.85^$$^	25.6%
7	Castor oil + 4-AP + Cromakalim	5 + 10	6.70 ± 0.50	2.80 ± 0.75	3.90 ± 0.35^$^	58.2%
8	Castor oil + GB + Loperamide	3 + 10	4.90 ± 0.39	1.40 ± 0.50	3.50 ± 0.90	71.4%
9	Castor oil + 4-AP + Loperamide	5 + 10	5 ± 1.73	1.31 ± 0.90	3.69 ± 0.45	73.8%
10	Castor oil + GB + Mc.Cr	3 + 150	5.83 ± 0.50	3.84 ± 0.37^$^	1.99 ± 0.36	34.1%
11	Castor oil + 4-AP + Mc.Cr	5 + 150	6.11 ± 0.49^$^	4.73 ± 0.31^$$^	1.38 ± 0.41^$^	22.5%
12	Castor oil + GB + Mc.Cr	3 + 300	5.56 ± 0.50^$^	3.06 ± 0.57^$$^	2.50 ± 0.62	44.9%
13	Castor oil + 4-AP + Mc.Cr	5 + 300	5.80 ± 0.46^$$^	4.07 ± 0.57^$$$^	1.73 ± 0.70^$^	29.8%

p.o. (per oral), Values shown are mean ± S.E.M. of 6 determinations.

^**#**^p <0.05 and ^**###**^p <0.001 show a comparison of group #2 and 3 with group #1.

*p <0.05, **p <0.01 and ***p <0.001show a comparison of group # 4 and 5 with group #1.

^$^p <0.05, ^$$^p <0.01 and ^$$$^p <0.001 show a comparison of group # 6–13 with their respective treatments without GB or 4-AP (One-way ANOVA followed by Dunnet’s test).

Further to assess its inhibitory effect on intestinal secretions, Mc. Cr was studied on castor oil-induced intestinal fluid accumulation in mice.

### Effect of *Matricaria chamomillla* on castor oil-induced intestinal fluid accumulation in mice

**Figure 1:**
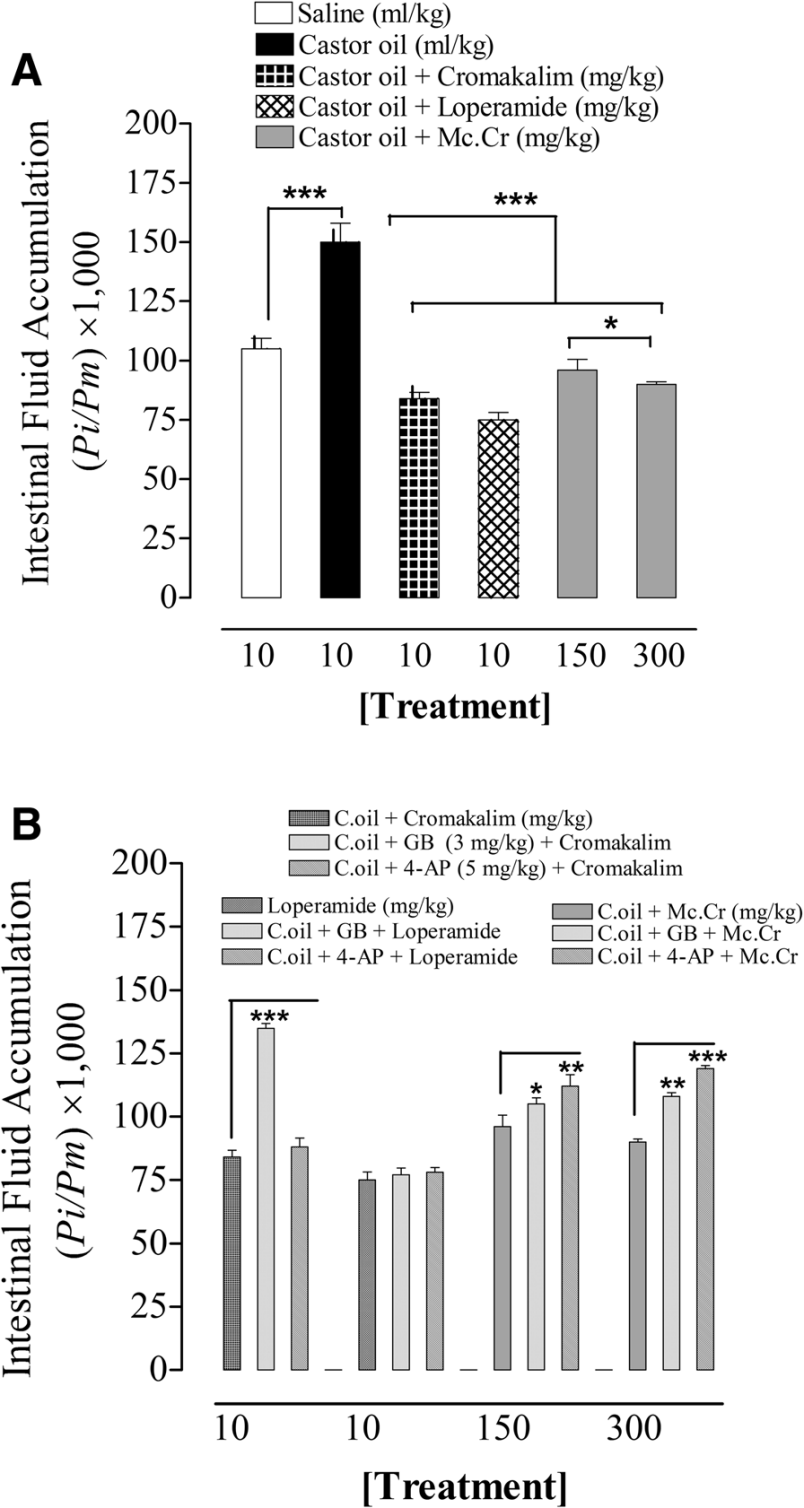
**Antidiarrhoeal and antisecretory effects of** ***Matricaria chamomilla*** **in mice.** The dose-dependent inhibitory effect of the crude extract of *Matricaria chamomilla* (Mc. Cr) on castor oil-induced fluid accumulation in the small intestine of mice, **(A)** without and **(B)** with glibenclamide (GB) or 4-aminopyridine (4-AP). Intestinal fluid accumulation is expressed as *Pi/Pm* × 1000, whereas *Pi* is the weight of the small intestine and *Pm* is the weight (in g) of the mouse. The values are mean ± s.e.m., n = 6. *p < 0.05, **p < 0.01 and ***p < 0.001 (Unpaired *t-*test or One-way ANOVA followed by Dunnett’s test).

In enteropooling assay, castor oil administration caused a substantial increase (*p* < 0.001) of fluid accumulation in mice with (*Pi/Pm)* × 1,000 value of 150 ± 8.0 when compared with saline administered group (105 ± 4.5). The administration of Mc. Cr to mice produced a significant (*p* < 0.001) reduction in castor oil-induced fluid accumulation at 150 and 300 mg/kg with respective (*Pi/Pm)* × 1,000 values of 96 ± 4.6 and 90 ± 1.2, similar to the effects cromakalim (84 ± 2.7) and loperamide (75 ± 3.2) at the doses of 10 mg/kg, respectively. When the inhibitory potential of Mc. Cr on castor oil-induced fluid accumulation was restudied in mice pretreated with GB or 4-AP, it was attenuated with resultant values of 105 ± 2.4 and 112 ± 4.9 vs. 96 ± 4.6 (effect of Mc. Cr without antagonist), respectively at 150 mg/kg, and also decreased with respective values of 108 ± 1.5 and 119 ± 1.2 vs. 90 ± 1.2 at 300 mg/kg. Similarly, the effect of cromakalim was significantly reduced in the presence of GB with resultant value of 135 ± 1.8 vs. 84 ± 2.7, while it remained unaltered in the presence of 4-AP. However, loperamide did not show sensitivity to GB or 4-AP (Figure 1). All (*Pi/Pm)* × 1,000 values are expressed as mean ± s.e.m. (*n* = 6).

### Acute toxicity test

The plant extract was well tolerated by the animals up to the tested oral dose of as high as 5 g/kg. No sign of acute toxicity like restlessness, seizures and piloerection was noticed over the period of observation (6 h) and there was no death recorded up to 24 h.

In order to investigate the presence of gut relaxant constitutes in Mc. Cr, which might be mediating its antidiarrhoeal and antisecretory activities, isolated rabbit jejunum was selected for further studies.

### Effect of *Matricaria chamomillla* on Rabbit Jejunum

**Figure 2:**
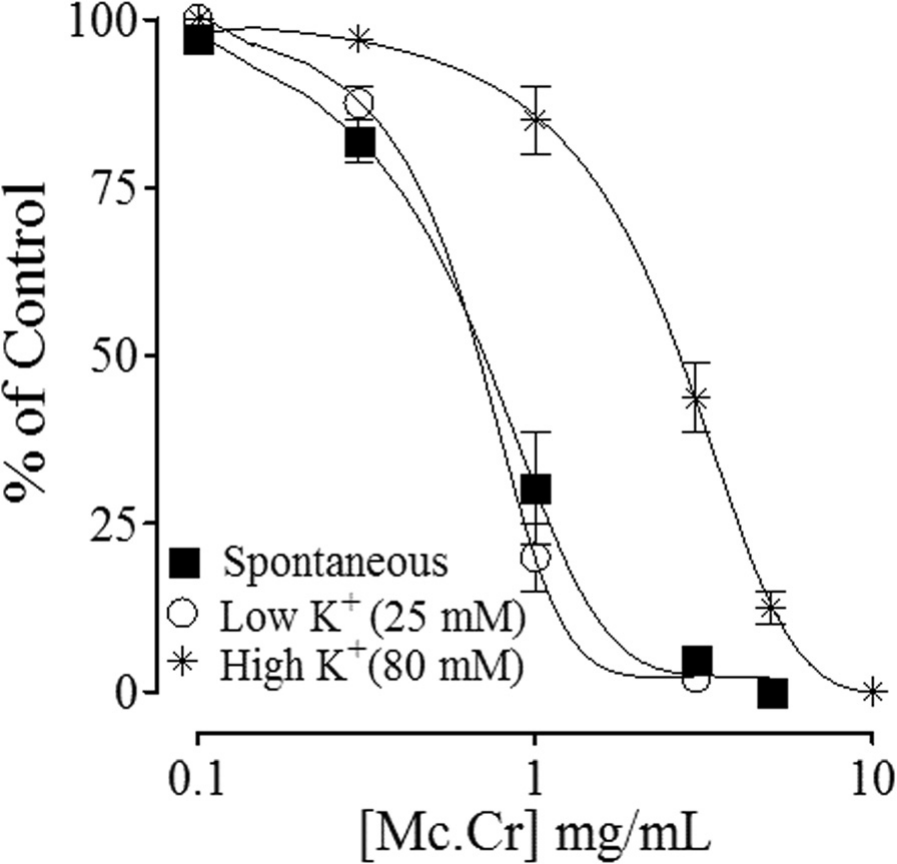
**The concentration-response inhibitory curves of the crude extract of** ***Matricaria chamomilla*** **(Mc. Cr) on spontaneous, low K** ^**+**^ **and high K** ^**+**^ **-induced contractions in isolated rabbit jejunum preparations.** The values shown are mean ± s.e.m. from 5–7 determinations.

**Figure 3:**
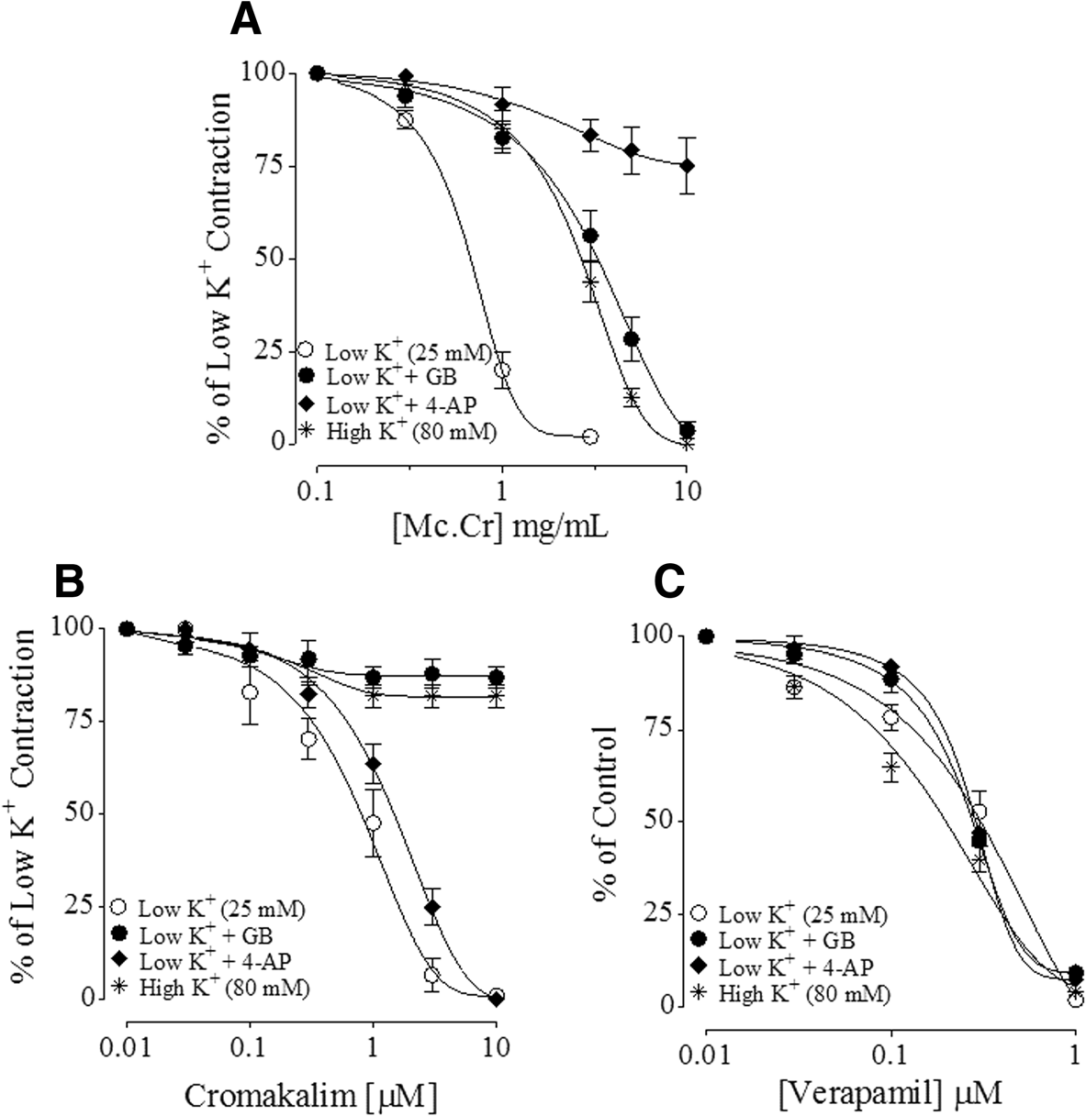
**Antispasmodic effect of** ***Matricaria chamomilla*** **against low K** ^**+**^ **(25 mM) contractions.** Concentration-response inhibitory curves showing comparison of **(A)** the crude extract of *Matricaria chamomilla* (Mc. Cr), **(B)** cromakalim and **(C)** verapamil in the absence and presences of 10 μM glibenclamide (GB) or 1 mM 4-aminopyridine (4-AP) against low K^+^ (25 mM)-induced contractions in isolated rabbit jejunum preparations. The values shown are mean ± s.e.m. from 4–6 determinations.

The plant extract inhibited potently the spontaneous and low K^+^-induced contractions with respective EC_50_ values of 0.89 mg/ml (0.63-1.19, 95% CI, n = 5) and 0.77 mg/ml (0.59-0.97, n = 7) compared to its effect (2.80 mg/ml (1.79-3.21, n = 6) on high K^+^-induced contractions as shown in Figure 2. When its effect on low K^+^-induced contractions was restudied in the presence glibenclamide (GB), it was significantly inhibited with resultant EC_50_ value of 3.09 mg/ml (2.21-3.76, n = 4) vs. 0.77 mg/ml (0.59-0.97, n = 7). In the presence 4-aminopyridine (4-AP), the relaxant effect of Mc. Cr was almost blocked with remaining maximum relaxation of only 29.73 ± 12.70% vs. 100% (n = 4-5) at the highest tested concentration of 10 mg/ml (Figure 3A).

Cromakalim also caused complete relaxation of low K^+^ with an EC_50_ value of 0.92 μM (0.56-1.34, n = 5), while it had negligible effect on high K^+^-induced contractions, producing only 27.19 ± 7% (n = 4) relaxation at the highest tested concentration of 10 μM. When its effect on low K^+^-induced contractions was studied in tissues pretreated with GB, it was significantly inhibited with remaining maximum relaxation of only 20.66 ± 4.29% vs. 100% (without GB) at the highest tested concentration (10 μM). However, there was no significant change in the relaxant effect of cromakalim [(1.10 μM (0.68-1.89, n = 5) vs. 0.92 μM (0.56-1.34, n = 4)] when reproduced in the presence of 4-AP, as shown in Figure 3B. Verapamil caused relaxation of high K^+^ and low K^+^-induced contractions at similar concentrations with respective EC_50_ values of 0.19 μM (0.12-0.28, n = 6) and 0.27 μM (0.20-0.37, n = 5). However, the relaxant effect of verapamil on low K^+^-induced contractions was found insensitive to GB and 4-AP (Figure 3C).

**Figure 4:**
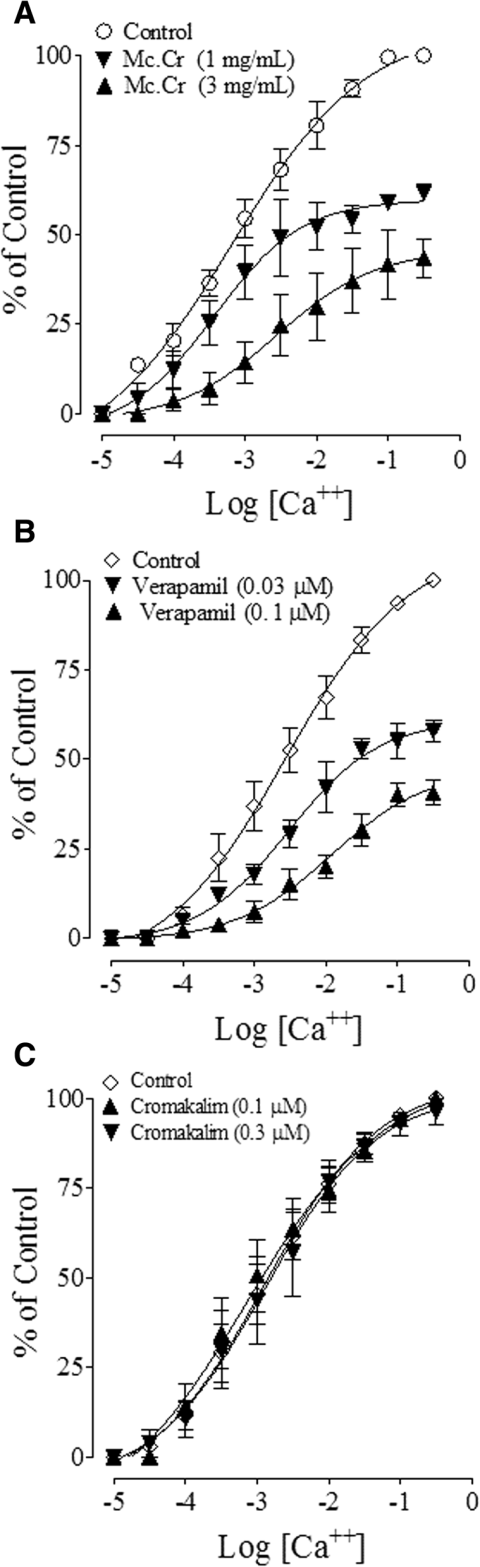
**Ca** ^**++**^ **antagonist effect o** ***f Matricaria chamomilla*** **against CaCl**_**2**_ **contractions.** The concentration-response curves of Ca^++^ constructed in the absence and presence of increasing concentration of **(A)** The crude extract of *Matricaria chamomilla* (Mc. Cr), **(B)** verapamil and **(C)** cromakalim in isolated rabbit jejunum preparations. The values shown are mean ± s.e.m. from 4–5 determinations.

The inhibitory effect of the plant extract was studied on high K^+^-induced contractions, to confirm if it exhibits Ca^++^ channel blockade-like activity at high doses. Interestingly the plant extract caused a rightward shift in the concentration response curves (CRCs) of Ca^++^ with the suppression of the maximum response at 1 and 3 mg/ml, respectively (Figure 4A). A positive control, verapamil also produced a rightward shift in the CRCs of Ca^++^and showed marked attenuation in the maximum response of Ca^++^ at applied concentrations of 0.03 and 0.1 μM (Figure 4B). Cromakalim was devoid of any effect in the CRCs of Ca^++^ (Figure 4C) as expected.

## Discussion

To validate the medicinal use of the crude extract of *Matricaria chamomilla* in hyperactive gut disorders, like diarrhoea and abdominal colic [1,6], this study was designed to determine the antidiarrhoeal, antisecretory and antispasmodic activities of the plant extract using the *in-vivo* and *in-vitro* assays. To study the insight into mechanisms isolated rabbit jejunum preparations were used.

In castor oil-induced diarrhoea and intestinal fluid accumulation models, Mc. Cr showed antidiarrhoeal and antisecretory activities at 150 and 300 mg/kg, similar to the effects of cromakalim [38] and loperamide [39], which are known for their spasmolytic, antidiarrhoeal and antisecretory activities. Castor oil is known to cause increased intestinal fluid contents and promotes diarrhoea indirectly through the effect of its active constituent, ricinoleic acid formed by the hydrolysis of oil [40], which changes the electrolytes and water transport [41] and generates massive contractions in transverse and distal colon [42]. The observed antidiarrhoeal and antisecretory effects of the plant extract appear to be mediated partly through the involvement of potassium channels activation which was evident by partial attenuation of these effects when reproduced in mice pretreated with glibenclamide (GB), an ATP-dependent K^+^ channel blocker [34] or 4-aminopyridine (4-AP), a voltage-dependent K^+^ channel blocker [35], thus, indicating the presence of gut inhibitory constituents in Mc. Cr which might me mediating its observed antidiarrhoeal and antisecretory effects.

The role of multiple types of physiological mediators, such as, acetylcholine, histamine, substance-P, cholecystokinins, prostaglandins and 5-hydroxytryptamine [43] and some ion channels like, K^+^ or Ca^++^, is well established in regulatory function of gastrointestinal system. Further, it has also been documented that most of the spasmolytic agents have therapeutic potential in diarrhoea by causing relaxation of the smooth muscle of the gut, in turn retain luminal fluid in the bowl [38,44].

It has been observed that most of the plant and plant-based test materials exhibit inhibitory effect through K^+^ channel activation or Ca^++^ channel blockade like mechanisms [30-33,45,46]. The use of low K^+^ (25 mM) and high K^+^ (80 mM)-induced depolarization in the tissues is usually carried out to distinguish K^+^ channel opening and Ca^++^ channel blocking like activities [32,33]. On the basis of presence of K^+^ channels and voltage dependent Ca^++^ channels in intestinal smooth muscles and epithelial cells [46], K^+^ channel openers (increase in K^+^ efflux) and Ca^++^ antagonists (inhibition of Ca^++^ entry) cause smooth muscle relaxation by decreasing intracellular free Ca^++^, through respective mechanisms of membrane hyperpolarization [38,44].

To assess whether the inhibitory effect of Mc. Cr was also mediated via similar mechanisms, it was tested on low and high K^+^-induced contractions in isolated rabbit jejunum, where it caused complete relaxation of low and high K^+^-induced contractions, being more potent against low K^+^. Cromakalim, a known ATP-dependent K^+^-channel opener [38], inhibited only low K^+^-induced contractions, while verapamil, a known Ca^++^ antagonist [44], was found equipotent against both low and high K^+^-induced contractions. These data show that the presence of spasmolytic constituents in *Matricaria chamomilla*, is likely to be responsible for its observed antidiarrhoeal and antisecretory activities in mice, mediating their effect primarily through K^+^ channel opening (KCO) along with weak Ca^++^ channel blockade (CCB) component.

To know the nature of K^+^ channels involved in KCO activity of Mc. Cr, the inhibitory CRCs of plant extract against low K^+^ were constructed in the absence and presence of GB or 4-AP. Interestingly, in line with *in vivo* findings in mice, the inhibitory effect of Mc. Cr was potently inhibited in the presence 4-AP compared to GB. This indicates that the KCO activity of plant extract predominantly involves voltage-dependent K^+^ channels along with ATP-sensitive K^+^ channels, which are abundantly present in intestinal smooth muscles and are also known for their inhibitory influence in hypermotile gut [47].

The concentration of K^+^ > 30 mM, regarded as high K^+^, is known to cause smooth muscle contractions through opening of voltage-dependent Ca^++^ channels [36]. Thus, a substance that inhibits high K^+^-provoked contractions is considered a blocker of Ca^++^ influx [48]. The Ca^++^ antagonist effect was confirmed when Mc. Cr, at slightly higher concentrations, shifted the CRCs of Ca^++^ to the right with suppression of the maximum effect, a typical characteristic of Ca^++^ antagonists [37], which are known for their antispasmodic, antisecretory and antidiarrhoeal activities [43,44]. These findings attest the presence of CCB-like spasmolytic continents in *Matricaria chamomilla* in addition to its primary effect as K^+^ channel opener. This study are not only provides an evidence to the medicinal use of *Matricaria chemomilla* in diarrhoea but also highlights the potential of this popular medicinal plant for the development of newer therapeutic options to treat hyperactive gut disorders, such as gut spasms and diarrhoea.

## Conclusions

These findings indicate that *Matricaria chamomilla* possesses antidiarrhoeal, antisecretory and antispasmodic activities mediated predominantly through K^+^ channels activation along with weak Ca^++^ channel antagonist like pathways, thus, providing an evidence to the medicinal use of *Matricaria chamomilla* in abdominal colic and diarrhoea.

## Abbreviations

Mc. Cr: Crude extract of *Matricaria chamomilla*
Low K^+^: K^+^ (25 mM)
High K^+^: K^+^ (80 mM)
GB: Glibenclamide
4-AP: 4-aminopyridine
EC_50_: Effective concentration producing 50% effect
CI: Confidence interval
ACh: Acetylcholine
CRCs: Concentration-response curves
p.o.: Per oral
i.p: Intraperitoneally
KCO: K^+^ channel opening
CCB: Ca^++^ channel blockade
